# Outcomes of a GnRH Agonist Trigger Following a GnRH Antagonist or Flexible Progestin-Primed Ovarian Stimulation Cycle

**DOI:** 10.3389/fendo.2022.837880

**Published:** 2022-05-19

**Authors:** Erkan Kalafat, Engin Turkgeldi, Sule Yıldız, Merve Dizdar, Ipek Keles, Baris Ata

**Affiliations:** ^1^ Division of Reproductive Endocrinology and Infertility, Department of Obstetrics and Gynecology, Koc University School of Medicine, Istanbul, Turkey; ^2^ Department of Statistics, Faculty of Arts and Sciences, Middle East Technical University, Ankara, Turkey; ^3^ Department of Obstetrics and Gynecology, Umraniye Teaching and Research Hospital, Istanbul, Turkey; ^4^ ART Fertility Clinics, Dubai, United Arab Emirates

**Keywords:** assisted reproduction, ovarian stimulation, progestin, agonist trigger, luteinising hormone, GnRH antagonist, Progestin primed ovarian stimulation, LH surge

## Abstract

A suggested explanation for the pituitary-suppressive effects of progestin-primed ovarian stimulation cycles (PPOS) is pituitary luteinizing hormone (LH) depletion with progestin exposure during the follicular phase. The GnRH agonist (GnRHa) trigger releases endogenous LH from the pituitary, and if the LH depletion theory is correct, the response to the agonist trigger would be dampened in PPOS cycles. In this study, we compared the performance of the GnRHa trigger after PPOS and GnRH antagonist ovarian stimulation cycles. All women who underwent ovarian stimulation with the GnRH antagonist or flexible PPOS (fPPOS) and received a GnRH agonist trigger were eligible for inclusion. Outcomes included number of metaphase-II (MII) oocytes retrieved per cycle, rates of empty follicle syndrome, maturation, fertilization, blastulation, and cumulative clinical pregnancy per stimulation cycle. During the screening period, there were 166 antagonists and 58 fPPOS cycles triggered with a GnRH agonist. Groups were matched for potential confounders using propensity score matching. Progestin-downregulated cycles had 19% high mature oocyte yield (median: 14 vs. 19 MII oocytes, P = 0.03). Cumulative ongoing pregnancy or live birth rates were estimated after matching for transferred embryo count, and rates were similar between GnRH antagonist and fPPOS group (57.0% vs. 62.1%, P = 0.68). However, the number of remaining blastocysts was higher in the fPPOS group (median: 5.0 vs. 6.0, P < 0.001). LH levels were higher in fPPOS cycles compared to GnRH antagonist cycles up to the trigger day (P < 0.001). After the GnRHa trigger, fPPOS cycles were associated with a steeper LH surge compared with antagonist cycles (P = 0.02). Higher endogenous gonadotropin levels through the stimulation period and an LH surge of higher magnitude following a GnRHa trigger suggest a milder pituitary suppression by fPPOS, which needs to be confirmed in larger samples. It appears that progestins do not deplete pituitary LH reserves and a GnRHa trigger is usable after PPOS in women with high ovarian reserve.

## Introduction

Gonadotropin-releasing hormone (GnRH) antagonists are the most commonly used drugs to achieve pituitary suppression in women undergoing ovarian stimulation. Progestins are recently being used to the same end in what is called progestin-primed ovarian stimulation (PPOS). PPOS seems similarly effective to GnRH antagonists in preventing premature ovulation (1). The advantages of PPOS are oral administration rather than injections and lower cost when a fresh transfer is not intended.

Despite the remarkable effectiveness of progestins in high and low responders for preventing premature ovulation, the exact mechanism of pituitary suppression remains elusive. Dozortsev et al. proposed that continuous progestin exposure before physiologic ovulation trigger may deplete pituitary luteinizing hormone (LH) reserves and desensitize GnRH receptors akin to GnRH agonist protocols (2). The mechanism proposed by Dozortsev et al. is a mere conjecture but may have important implications for clinical practice. PPOS cycles can be preferentially used for anticipated high responders to be followed by a GnRH agonist trigger and freeze-all approach to prevent ovarian hyperstimulation syndrome and maintain live birth rates (3). If PPOS indeed depletes pituitary LH reserve, then the response to a GnRH agonist trigger can be dampened leading to a decreased mature oocyte yield. While the performance of the GnRH agonist trigger has been indirectly evaluated in conventional PPOS cycles, i.e., where progestins are started simultaneously with gonadotropins, it is less well known in flexible PPOS (fPPOS), i.e., where progestins are started later than gonadotropins when the follicles reach a certain level of development as in flexible GnRH antagonist cycles (4). We compared LH levels, oocyte yield, and clinical outcomes following a GnRH agonist trigger in GnRH antagonist and fPPOS cycles.

## Methods

This was a retrospective cohort study using anonymized patient data. The Koc University Clinical Research Ethics Committee approved the study protocol. All women were treated at the Assisted Reproduction Unit of the Koc University Hospital between August 2016 and May 2021. All women who underwent ovarian stimulation with the GnRH antagonist or fPPOS and received a GnRH agonist trigger were eligible for inclusion. Women with decreased ovarian reserve, who received human chorionic gonadotropin (hCG) trigger, and who underwent oocyte cryopreservation cycles were excluded.

Ovarian pathology was excluded with a baseline ultrasound scan on the 2nd/3rd day of menstruation. All women underwent ovarian stimulation with a 225- or 300-IU daily dose of gonadotropins (recombinant follicle-stimulating hormone; GONAL-f, Merck, Kenilworth, NJ, USA, or human menopausal gonadotropin; Merional, IBSA, Lugano, Switzerland). In fPPOS cycles, medroxyprogesterone acetate (MPA) 10 mg/day p.o. was started when the leading follicle **≥**14 mm or serum estradiol (E_2_) >200 ng/ml, whichever came first. GnRH antagonist 250 mcg/day subcutaneously (Cetrotide, Merck) was also started in a flexible fashion like MPA (4). In both protocols, MPA and GnRH antagonists were continued until (including) the day of the ovulation trigger. Oocyte maturation was triggered with 0.2 mg triptorelin acetate, when the leading follicle size was greater than 17 mm with the majority of others >14 mm, followed by oocyte retrieval 36 h later. Embryology laboratory procedures including denudation, intracytoplasmic sperm injection, embryo culture, oocyte/embryo vitrification and thawing, luteal phase support, and endometrial preparation for frozen embryo transfers are described in our former publications (5–7).

Outcomes included number of metaphase-II (MII) oocytes retrieved per cycle, rates of empty follicle syndrome, maturation, fertilization, blastulation, and cumulative clinical pregnancy per stimulation cycle. Empty follicle syndrome was defined as failure to collect any oocytes in an oocyte pick-up procedure. The maturation rate was calculated as the ratio of MII to total oocyte count. The fertilization rate was calculated as the ratio of two pronuclear embryos to MII oocytes. The blastulation rate was calculated as the ratio of blastocyst to two pronuclear embryos. Cumulative clinical pregnancy was defined as an ongoing pregnancy above 10 weeks’ gestation until exhaustion of all available embryos or within 6 months of the original oocyte stimulation cycle. Moreover, we investigated serum LH levels throughout stimulation and 12 h after the administration of the GnRH agonist trigger.

### Statistical Analysis

Continuous variables with a normal distribution were presented with mean and standard deviation, while skewed distributions were presented with median and 25th–75th percentiles. Age and initial gonadotropin dose were identified as potential confounders, and groups were checked for imbalances regarding these variables with propensity scores. Then groups were matched using exact matching on female age and nearest matching on gonadotropin dose. Age was categorized into five categories (<30, 30–35, 35–38, 38–40, >40 years) to help find matches. After propensity score matching of both groups, the balance was checked visually using propensity score histograms and mean standardized differences were checked to be under 0.1 points. For cumulative pregnancy outcomes, matching was also performed on number of transferred embryos. The outcomes were compared between fPPOS and GnRH antagonist groups with mixed-effect generalized models using match identifiers and patient identifiers (repeat cycles) as random effects. Log-binomial link function was used for binary outcomes, and log-Poisson link function was used for count data (MII oocyte count, follicle count). Outcomes were reported as rate ratios with 95% confidence intervals. Violin plots were used for graphical representation of the results. All analyses were conducted with R for Statistical Computing Software (v.4.0.4).

## Results

During the screening period, there were 166 antagonist and 58 fPPOS cycles triggered with a GnRH agonist. There were no statistically significant differences between antagonist and fPPOS cycles regarding female age (median: 31.0 vs. 30.5 years, respectively, P = 0.10), body mass index (median: 24.6 vs. 22.9 kg/m^2^, respectively, P = 0.10), and serum AMH levels (median: 4.6 vs. 5.0 ng/ml, respectively = 0.71) (**Table 1**). Women undergoing fPPOS were more often stimulated with rFSH compared to the GnRH antagonist, despite the difference being short of statistical significance (74.1 vs. 60.8%, P = 0.10). After propensity score matching for female age and initial gonadotropin dose, the groups were balanced for these variables with mean standardized differences smaller than 0.1 (**Table 2**, **Supplementary Figure 1**). Residual imbalances remained regarding gonadotropin type, but the sample size did not allow controlling for these variables *via* propensity score matching. Instead, regression analyses were adjusted (**Supplementary Figure 2**) for these variables to check for consistency of the estimates.

**Table 1:** Baseline and ovarian stimulation characteristics.

Variables	GnRH antagonist (n = 166)	Flexible PPOS (n = 58)	*P^a^*
Female age in years	31.0 (29.0–34.75)	30.5 (28.0–33.0)	.10
BMI in kg/m^2^	24.6 (21.8–27.4)	22.9 (21.5–25.7)	.10
Serum AMH levels in ng/mL	4.6 (3.1–6.4)	5.0 (3.6–6.4)	.71
Indication for ovarian stimulation			
- Anovulatory (PCO)	49 (29.5)	15 (25.7)	.60
- Endometriosis	7 (4.2)	2 (3.4)	.80
- Uterine factor	6 (3.6)	5 (8.6)	.13
- Tubal factor	6 (3.6)	2 (3.4)	.95
- Male factor (any)	50 (30.1)	18 (31.0)	.90
- Unexplained	48 (28.9)	16 (27.6)	.85
Gonadotropin type			.07
- rFSH	101 (60.8)	43 (74.1)	
- HMG	65 (39.2)	15 (25.9)	
Initial gonadotrophin dose in IU/day	300.0 (225.0–300.0)	300.0 (225.0–300.0)	.15
Duration of stimulation	10.0 (8.0–10.0)	9.0 (8.0–9.0)	<0.001
Serum E_2_ levels in pg/mL at maturation trigger	3008 (2391–3959)	4034 (2845–5993)	<0.001
Follicle larger than 14 mm at maturation trigger	9.0 (7.0–13.0)	11.0 (8.0–16.0)	.02
Leading follicle size in mm at maturation trigger	20.0 (19.0–22.0)	20.0 (19.0–21.0)	.17

a Wilcoxon-signed rank test, t-test, or chi-squared test where appropriate.

BMI, body mass index; PCO, polycystic ovaries; rFSH, recombinant follicle stimulating hormone; HMG, human menopausal gonadotrophin.

Values are presented as median and interquartile range or count and percentage of total.

**Table 2:** Baseline and stimulation characteristics after propensity score matching for female age and initial gonadotropin dose.

Variables	GnRH antagonist (n = 116)	Flexible PPOS cycles (n = 58)	*P^a^*
Female age in years	30.0 (28.0–34.0)	30.5 (28.0 0 33.0)	.59
BMI in kg/m^2^	24.2 (21.6–27.2)	22.9 (21.5–25.7)	.64
Serum AMH levels in ng/mL	4.5 (2.8–6.5)	5.0 (3.6–6.4)	.54
Indication for ovarian stimulation			
- Anovulatory (PCO)	28 (24.1)	15 (25.9)	.80
- Endometriosis	7 (6.0)	2 (3.4)	.46
- Uterine factor	5 (4.6)	5 (8.6)	.24
- Tubal factor	5 (4.3)	2 (3.4)	.78
- Male factor (any)	35 (30.1)	18 (31.0)	.90
- Unexplained	36 (31.0)	16 (27.6)	.63
Gonadotrophin type			.011
- rFSH	63 (54.3)	43 (74.1)	
- HMG	53 (45.7)	15 (25.9)	
Initial gonadotropin dose in IU/day	300.0 (225.0–300.0)	300.0 (225.0–300.0)	.85
Duration of stimulation	9.0 (8.0–10.0)	9.0 (8.0–9.0)	.008
Serum E_2_ levels in pg/mL at maturation trigger	3434 (2948–4734)	4034 (2845–5993)	.17
Follicle larger than 14 mm at maturation trigger	9.0 (7.0–13.0)	11.0 (8.0–16.0)	.062
Leading follicle size in mm at maturation trigger	20.0 (19.0–22.0)	20.0 (19.0–21.0)	.016

^a^Generalized estimating equations using match ID as clusters.

BMI, body mass index; PCO, polycystic ovaries; rFSH, recombinant follicle stimulating hormone; HMG, human menopausal gonadotrophin; HP-uFSH, highly purified urinary follicle stimulating hormone, PPOS: progestin primed ovarian stimulation.

Values are presented as median and interquartile range or count and percentage of total.

**Figure 1:**
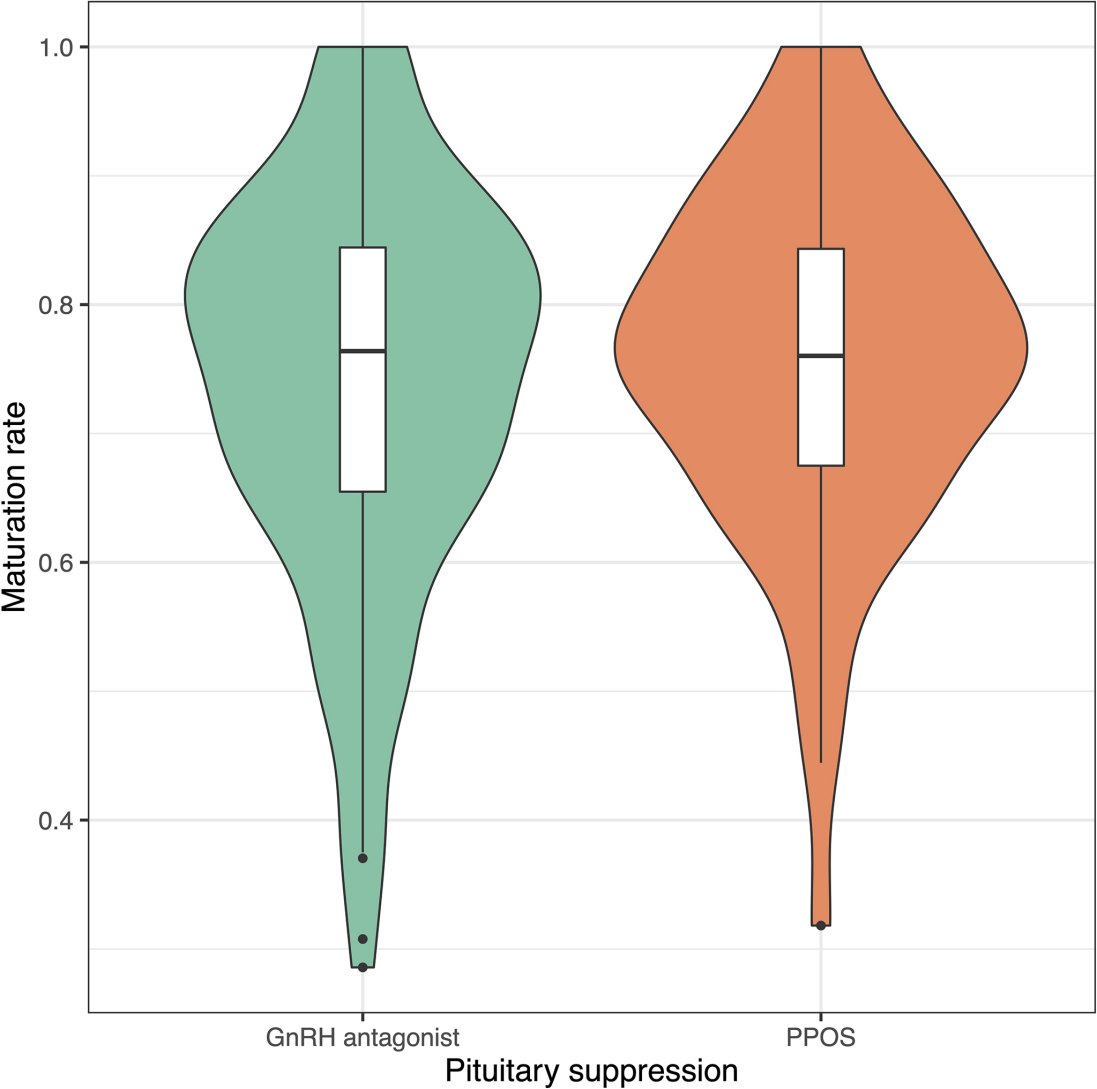
Violin plots showing maturation rate in cycles suppressed with GnRH antagonists (green) and progestins (orange). Colored shaded areas show the distribution density. Lower and upper bounds of box plots show the interquartile range, and horizontal dash shows the median. Black dots show potential outliers. GnRH, gonadotrophin-releasing hormone; PPOS, progestin-primed ovarian stimulation.

**Figure 2:**
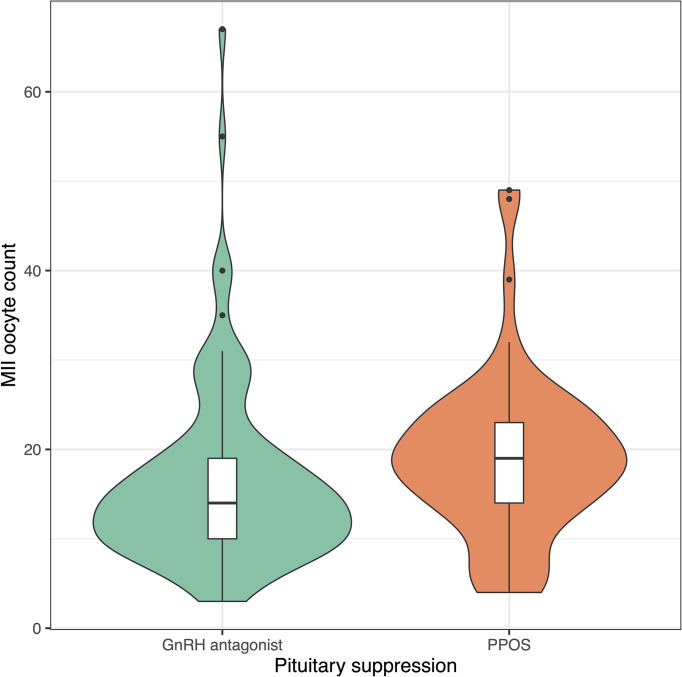
Violin plots showing metaphase-II oocyte count in cycles suppressed with GnRH antagonists (green) and progestins (orange). Colored shaded areas show the distribution density. Lower and upper bounds of box plots show the interquartile range and horizontal dash shows the median. Black dots show potential outliers. GnRH, gonadotrophin-releasing hormone; PPOS, progestin-primed ovarian stimulation.

**Figure 3:**
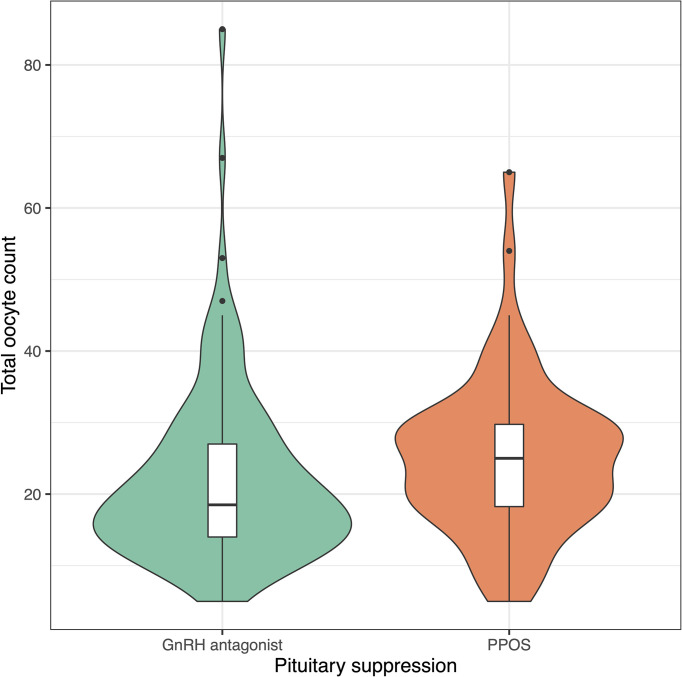
Violin plots showing total oocyte count in cycles suppressed with GnRH antagonists (green) and progestins (orange). Colored shaded areas show the distribution density. Lower and upper bounds of box plots show the interquartile range, and horizontal dash shows the median. Black dots show potential outliers. GnRH, gonadotrophin releasing hormone; PPOS, progestin primed ovarian stimulation.

Progestin-downregulated cycles had 19% high mature oocyte yield (median: 14 vs. 19 MII oocytes, P = 0.03), and the difference persisted after adjusting for gonadotropin type (**Table 3**). There were no differences between GnRH antagonist and fPPOS cycles regarding maturation (mean difference: 0.7%, P = 0.67), fertilization (mean difference: -0.03%, P = 0.21), and blastulation rate (mean difference: 0.02%, P = 0.51) of collected oocytes. Thus, the increased oocyte yield also translated to increased blastocyst yield (median: 6 vs. 8 blastocysts, P = 0.01). There were no significant changes in the estimates after adjusting for gonadotropin type (**Table 3**). Cumulative ongoing pregnancy or live birth rates were estimated after matching for **(Supplementary Figure 2)** transferred embryo count (median 1.0 vs. 1.0, P = 0.47), and rates were similar between the GnRH antagonist and the fPPOS group (56.0% vs. 62.1%, P = 0.65) (**Table 3**). However, the number of remaining blastocysts was higher in the fPPOS group (median: 5.0 vs. 6.0, P < 0.001). Violin plots depicting the maturation rate (**Figure 1**), MII oocyte yield (**Figure 2**), and total oocyte yield (**Figure 3**) are available.

**Table 3:** The association of pituitary suppression method with oocyte yield parameters in propensity score matched cohort.

Outcome	GnRH antagonist (n = 116)	Flexible PPOS (n = 58)	RR or MD (95% CI)	*P^a^*	aRR or aMD (95% CI)*^b^*	*P^a^*
MII oocyte count	14.0 (10.0–18.0)	19.0 (14.0–23.0)	1.19 (1.02 to 1.38)	.032	1.21 (1.12 to 1.30)	<.001
Total oocyte count	20.0 (14.0–27.0)	25.0 (18.0–30.0)	1.19 (1.10 to 1.29)	<.001	1.18 (1.10 to 1.26)	<.001
Maturation rate	75.0% (64.3–83.3)	76.0% (67.5–84.3)	0.7% (-2.5 to 3.8)	.67	0.4% (-2.8 to 3.5%)	.82
Empty follicle syndrome	0 (0.0)	0 (0.0)	NE	NE	NE	NE
Fertilization rate	75.0% (62.4–85.9%)	75.0% (60.3–80.0%)	-0.03% (-0.08 to 0.02)	.21	-0.04% (-0.10 to 0.01)	.12
Blastocyst count	6.0 (3.0–9.0)	8.0 (4.0–12.0)	1.33 (1.06 to 1.69)	.011	1.27 (1.14 to 1.43)	.012
Blastulation rate	53.8% (36.0–66.8%)	53.8% (35.0–77.3%)	0.02% (-0.05 to 0.10)	.51	-0.01 (-0.08 to 0.07)	.88
Cumulative ongoing pregnancy or livebirth*^c^*	65/116 (56.0)	36/58 (62.1)	1.10 ( 0.72 to 1.64)	.65	1.06 (0.70 to 1.59)	.78
Remaining blastocysts	5.0 (3.0–8.0)	6.0 (3.0–11.0)	1.28 (1.14 to 1.45)	<.001	1.24 (1.16 to 1.51)	<.001

^a^Mixed-effect linear or Poisson regression using match ID and patient ID as random intercepts.

^b^Adjusted for gonadotropin type.

^c^An ongoing pregnancy over 12 weeks’ gestation or live birth using embryos from the same stimulation cycle.

MII, metaphase II; PPOS, progestin primed ovarian stimulation; NE, not estimable; RR, risk ratio; MD, mean difference; CI, confidence interval.

**Figure 4:**
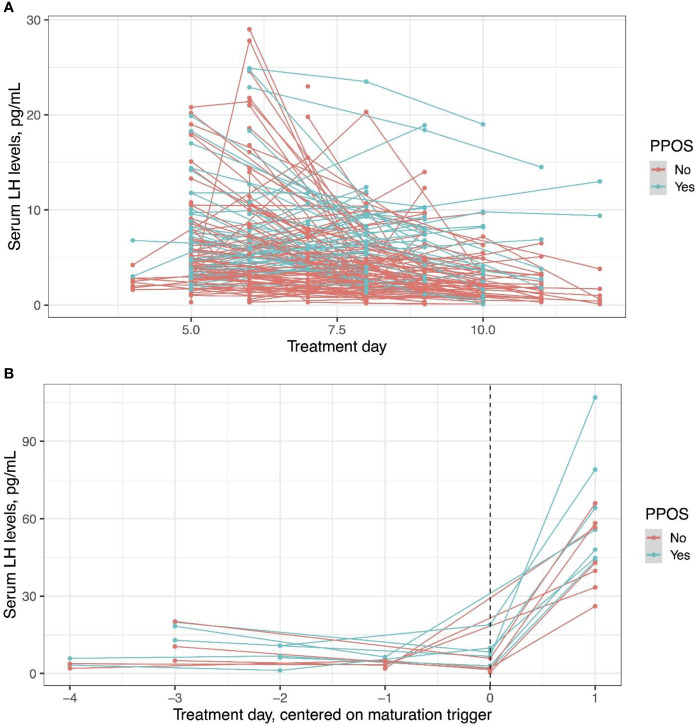
Serum luteinizing hormone levels in progestin-primed and antagonist cycles before the trigger day **(A)** and after the trigger day **(B)**. An agonist trigger was associated with higher luteinizing hormone levels throughout the cycle (P < 0.001) and a steeper luteinizing hormone surge in progestin-primed cycles compared with antagonist cycles (*P* = 0.02). Dots show individual data points and lines how the trajectory of change for individual patients.

LH levels measured prior to trigger was assessed in the whole cohort, and LH response to the agonist trigger was assessed in a limited cohort, who had post-trigger LH measurements available, seven women in fPPOS and eight in GnRH antagonist groups. LH levels were higher in fPPOS cycles compared to GnRH antagonist cycles up to the trigger day (**Figure 4A**, P < 0.001). After the GnRH agonist trigger, the change in serum LH levels was significantly different between fPPOS and GnRH antagonist cycles and the agonist trigger was associated with a steeper LH surge in fPPOS compared with antagonist cycles (P = 0.02, **Figure 4B**).

## Discussion

Higher endogenous gonadotropin levels through the stimulation period and an LH surge of higher magnitude following a GnRH agonist trigger provided by fPPOS could have probably led to the higher number of oocytes collected than the flexible GnRH antagonist protocol in women with a high ovarian reserve. Maturation, fertilization, and blastulation rates per collected oocyte were similar with the two protocols, leading to more usable blastocysts being available with the fPPOS and GnRH agonist trigger combination than the GnRH antagonist and GnRH agonist trigger combination. This probably can lead to higher cumulative live birth rates with fPPOS in this group of women.

A higher proportion of women were stimulated with rFSH in the fPPOS group than in the GnRH antagonist group. Arguably, this could have contributed to the difference in oocyte numbers between the two study groups, since rFSH provides a higher number of oocytes than hMG in GnRH antagonist cycles (8). Yet, analyses adjusted for the type of gonadotropin suggest that the effect of fPPOS on oocyte yield is independent of gonadotropin type. Moreover, results of the present study are similar with our prior study comparing the two protocols between oocyte donors, all of which were stimulated with rFSH (4). Oocyte donors stimulated with the fPPOS had produced more oocytes, which performed as well as oocytes collected from GnRH antagonist-stimulated cycles in terms of fertilization, blastulation, and live birth rates (4). One possible explanation of fPPOS providing a higher ovarian response can be milder suppression of the pituitary gonadotropin secretion by MPA than cetrorelix. Thus, endogenous gonadotropins could be contributing to ovarian stimulation. Indeed, in the present study, LH levels throughout the stimulation period were significantly higher in the fPPOS group than in the GnRH antagonist group. Moreover, pituitary response to the GnRH agonist trigger was stronger as reflected by higher post-trigger increase in fPPOS cycles. These theories generated by our observations require validation in adequately sized trials.

In conventional PPOS, serum gonadotropin levels throughout the stimulation period or after a GnRH agonist trigger are similar with ovarian stimulation using GnRH analogues (1). Likewise, oocyte yield also seems similar (1). Whether fPPOS, which provides higher endogenous gonadotropin levels and more oocytes than GnRH antagonist protocol in women with high ovarian reserve, also provides a higher oocyte yield than conventional PPOS remains to be seen. While we did not observe such a benefit of fPPOS in our retrospective study in women with decreased ovarian reserve (7), the limited potential of their ovaries could have prevented demonstration of a potential benefit. A comparison of the two PPOS protocols in women with high ovarian reserve is needed.

The exact mechanism of pituitary suppression by progestins is still unclear (1). Dozortsev et al. suggested that continuous stimulation of pituitary gonadotrophs by low-level progestin exposure through the follicular phase would deplete LH stores and prevent a spontaneous surge (2). Our observations do not lend credit to this theory. LH surge after GnRH agonist trigger had a higher magnitude in fPPOS cycles than in GnRH antagonist cycles. If progestin exposure depleted LH in the gonadotrophs, the magnitude of LH surge would have been lower. While the number of our observations is limited, such a deficit should have been still evident if there were such a mechanism affecting every cycle. On the contrary, Chen et al. reported similar post-GnRH agonist trigger LH levels between women who underwent a conventional PPOS or a GnRH antagonist protocol, suggesting that even longer exposures to progesterone throughout the follicular phase did not deplete LH in gonadotrophs (9). Our and others’ observations are also inconsistent with the theory that progesterone desensitizes GnRH receptors as a single shot of the GnRH agonist can trigger an endogenous LH surge in either a conventional PPOS, where progestins are started simultaneously with gonadotropins (10–13), or an fPPOS, where progestins are started later in the follicular phase (4).

Another question brought about by the effectiveness of the fPPOS protocol is whether progesterone alone is an ovulation trigger as suggested by Dozortsev et al. Women are started progestins toward the mid-follicular phase in the fPPOS protocol, when circulating estradiol levels are higher than preovulatory levels in a natural cycle with monofollicular development (4). Yet, progestins do not trigger ovulation in high or poor responders (4, 6). Possible explanations are progesterone not being a trigger of ovulation or the dose provided by fPPOS being insufficient to trigger ovulation.

The present study has the usual limitations of retrospective design, most important of which is the risk of selection bias. Propensity score matching decreases this risk for known prognostic factors, and the use of a multivariate regression model helps to address residual confounding. However, it is reassuring that our results were consistent with another retrospective study conducted by our team but in a different setting (4). Still, these should be regarded as hypothesis generating rather than conclusive.

In conclusion, our results suggest that in an fPPOS protocol, a GnRH agonist trigger functions as well as or better than in a GnRH antagonist protocol. More descriptive studies and clinical trials are required to elucidate the exact mechanism of pituitary suppression by PPOS and the best protocol.

## Data Availability Statement

The raw data supporting the conclusions of this article will be made available by the authors, without undue reservation.

## Ethics Statement

The studies involving human participants were reviewed and approved by the Koc University Ethics Committee. Written informed consent for participation was not required for this study in accordance with the national legislation and the institutional requirements.

## Author Contributions

Conception and design, BA, EK. Data acquisition, MD, IK, ET, SY. Analysis, EK. Drafting, BA, EK. Critical revision, BA, EK, ET, SY, IK. All authors contributed to the article and approved the submitted version.

## Conflict of Interest

The authors declare that the research was conducted in the absence of any commercial or financial relationships that could be construed as a potential conflict of interest.

## Publisher’s Note

All claims expressed in this article are solely those of the authors and do not necessarily represent those of their affiliated organizations, or those of the publisher, the editors and the reviewers. Any product that may be evaluated in this article, or claim that may be made by its manufacturer, is not guaranteed or endorsed by the publisher.

## Supplementary Material

Supplementary Figure 1 Propensity score histograms in flexible progestin primed ovarian stimulation (treated) and antagonist (control) cycles. Female age was categorised into <30, 30-35, 35-38, 38-40, >40 years to facilitate matching. Propensity score matching was performed on initial gonadotropin dose and female age.

Supplementary Figure 2 Propensity score histograms in flexible progestin primed ovarian stimulation (treated) and antagonist (control) cycles. Female age was categorised into <30, 30-35, 35-38, 38-40, >40 years to facilitate matching. Propensity score matching was performed on initial gonadotropin dose, female age, and transferred embryo count.
