# Supplementary figures and images for: Outcomes of a GnRH Agonist Trigger Following a GnRH Antagonist or Flexible Progestin-Primed Ovarian Stimulation Cycle
Source: Front Endocrinol (Lausanne). 2022 May 19;13:837880. doi: 10.3389/fendo.2022.837880 (PMC9161281; doi:10.3389/fendo.2022.837880)

**Raw Treated**

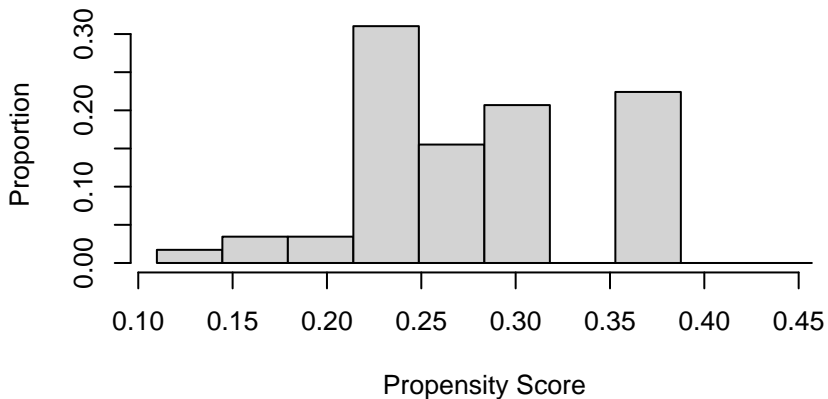

**Matched Treated**

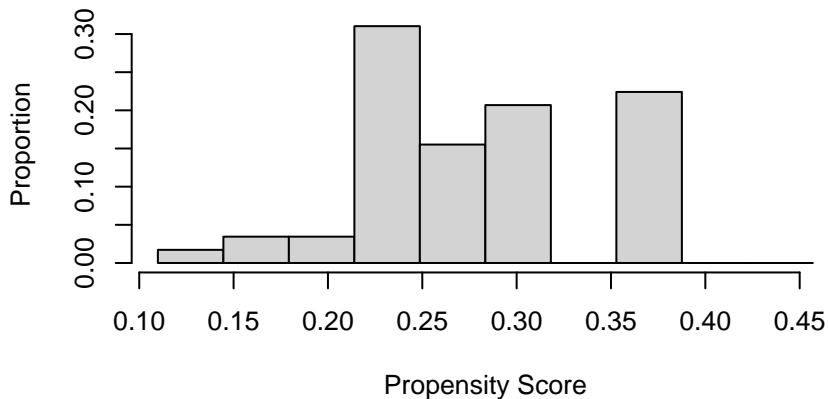

**Raw Control**

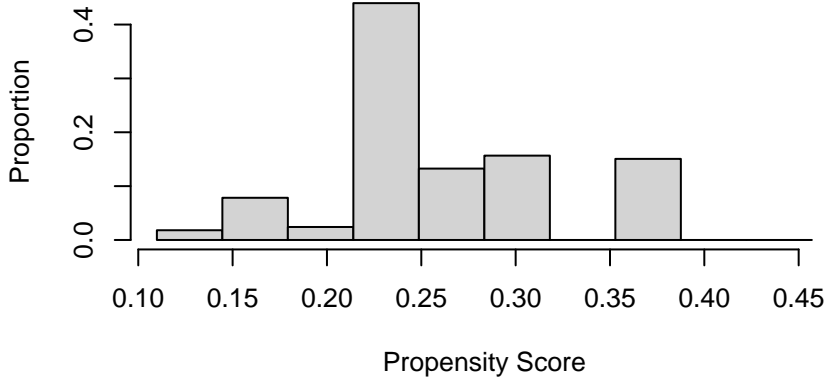

**Matched Control**

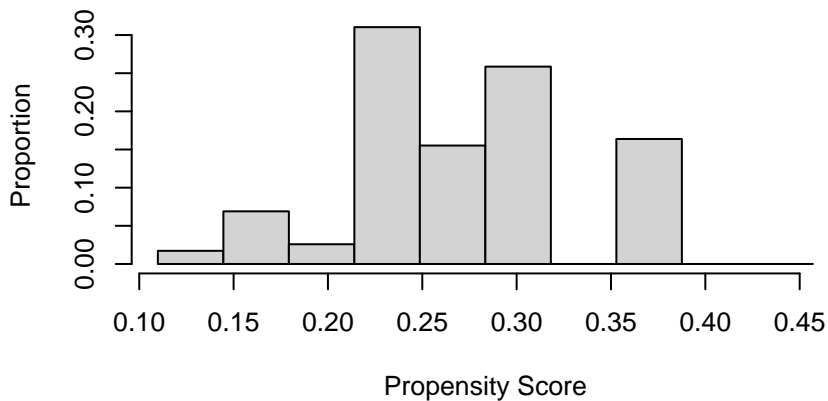

Supplement: Supplementary Figure 1 — Propensity score histograms in flexible progestin primed ovarian stimulation (treated) and antagonist (control) cycles. Female age was categorised into <30, 30-35, 35-38, 38-40, >40 years to facilitate matching. Propensity score matching was performed on initial gonadotropin dose and female age. [file DataSheet_1.pdf]

**Raw Treated**

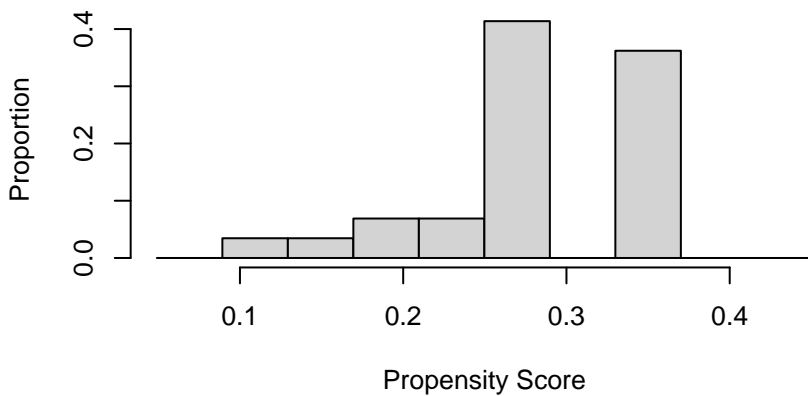

**Matched Treated**

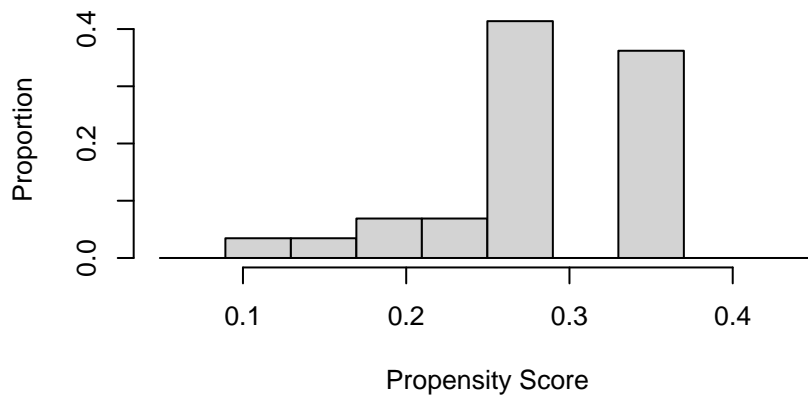

**Raw Control**

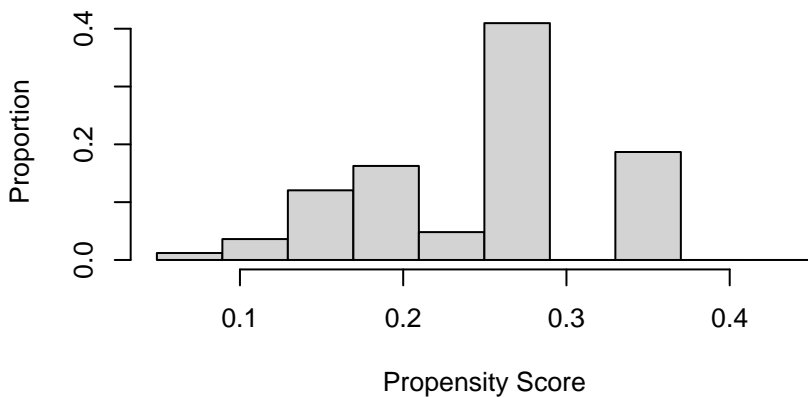

**Matched Control**

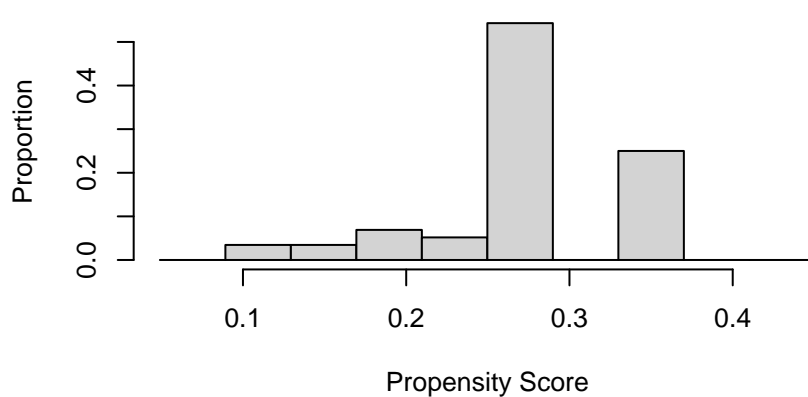

Supplement: Supplementary Figure 2 — Propensity score histograms in flexible progestin primed ovarian stimulation (treated) and antagonist (control) cycles. Female age was categorised into <30, 30-35, 35-38, 38-40, >40 years to facilitate matching. Propensity score matching was performed on initial gonadotropin dose, female age, and transferred embryo count. [file DataSheet_2.pdf]
